# Prediction of Three-Dimensional Downward Flame Spread Characteristics over Poly(methyl methacrylate) Slabs in Different Pressure Environments

**DOI:** 10.3390/ma9110948

**Published:** 2016-11-22

**Authors:** Kun Zhao, Xiao-Dong Zhou, Xue-Qiang Liu, Lei Lu, Zhi-Bo Wu, Fei Peng, Xiao-Yu Ju, Li-Zhong Yang

**Affiliations:** 1 State Key Laboratory of Fire Science, University of Science and Technology of China, Hefei 230027, China; zk008@mail.ustc.edu.cn (K.Z.); zxd@ustc.edu.cn (X.-D.Z.); wuzhibo@mail.ustc.edu.cn (Z.-B.W.); pfei@ustc.edu.cn (F.P.); ustcjxy@mail.ustc.edu.cn (X.-Y.J.); 2 China-Hemp Research Center, Beijing 100000, China; lxq@tsinghua.edu.cn; 3 Hainan Nuclear Power Co., Ltd., Haikou 570100, China; lulei@hnpc.cc; 4 Collaborative Innovation Center for Urban Public Safety, Hefei 230027, China

**Keywords:** downward flame spread, three-dimensional, poly(methyl methacrylate) (PMMA), pressure, thermal transfer

## Abstract

The present study is aimed at predicting downward flame spread characteristics over poly(methyl methacrylate) (PMMA) with different sample dimensions in different pressure environments. Three-dimensional (3-D) downward flame spread experiments on free PMMA slabs were conducted at five locations with different altitudes, which provide different pressures. Pressure effects on the flame spread rate, profile of pyrolysis front and flame height were analyzed at all altitudes. The flame spread rate in the steady-state stage was calculated based on the balance on the fuel surface and fuel properties. Results show that flame spread rate increases exponentially with pressure, and the exponent of pressure further shows an increasing trend with the thickness of the sample. The angle of the pyrolysis front emerged on sample residue in the width direction, which indicates a steady-burning stage, varies clearly with sample thicknesses and ambient pressures. A global non-dimensional equation was proposed to predict the variation tendency of the angle of the pyrolysis front with pressure and was found to fit well with the measured results. In addition, the dependence of average flame height on mass burning rate, sample dimension and pressure was proposed based on laminar diffusion flame theory. The fitted exponent of experimental data is 1.11, which is close to the theoretical value.

## 1. Introduction

Poly(methyl methacrylate) (PMMA) is widely used as a building material due to its excellent clarity and intensity. However, it easily becomes soft under heat and has a high flammability potential (Limited Oxygen Index = 18). Under a certain external heat flux, PMMA begins to bubble and many combustible gases will form. Once the temperature and concentration of the gases exceed the critical values, solid PMMA will be ignited. After ignition, the flame may spread in different directions, which may cause serious damage. Thus, prediction of flame spread rate is of great practical importance from a fire protection point of view. In a real-fire scenario, prediction of downward flame spread over solid combustibles is a complicated problem since it is not only related with the thermal properties of virgin material but also changes with environmental conditions, such as temperature, pressure, wind velocity and humidity. All these parameters have significant influences on heat and mass transfer processes, which are predominant in flame spread mechanisms. A number of experiments and simulations on flame spread over solid combustibles have been performed. The dependence of flame spread characteristics on related physical and ambient conditions has also been proposed [1,2,3,4,5,6,7,8,9,10,11,12,13,14].

Flame spread rate was one of the most studied parameters in previous studies. Numerical simulation is an important method investigating fire spread mechanisms since it gives the detailed information. Most theoretical models of downward flame spread reported in the literature can be classified as two-dimensional (2-D) heat transfer models [1,3,4,5,8,9,12]. De Ris [1] firstly presented an integrated theoretical description of flame spread rate over thin sheet and a semi-infinite fuel bed. A significant difference was found between the two sizes of simulation objects. Fernandez-Pello and Williams [3] optimized the model of gas phase flow and provided a detailed description of the dependence of flame spread rate on ambient temperature, pressure, oxygen concentration, gravity acceleration and sample thickness. In addition, Frey and T’ien [4] proposed that the dependence of flame spread rate on pressure over a thermal thin slab away from the extinct limit was slight and linearly proportional to oxygen concentration. Later, Delichatsios [8,9] developed a new energy balance in consideration of chemical-kinetic effects, external heat fluxes and reradiation losses. Altenkirch et al. [5] proposed a dimensionless flame spread rate for thermally thin paper sheets with Damkohler under different oxygen concentrations and pressures. Recently, Pujol and Comas et al. [15,16] derived analytical expression of flame front spread by focusing on the gas-phase equations, which was much more simplified and accurate. Leventon et al. [17] developed a comprehensive model to predict time to ignition and mass burning rate by coupling ThermaKin with empirical model of heat transfer. In general, the common conclusion is that flame spread rate for PMMA is closely related with sample thickness.

Apart from the theoretical predictions of flame spread rate mentioned above, many investigators have concentrated on experimental and semi-empirical research method [2,7,10,11,12,13,14,18,19]. Ayani et al. [10] provided an empirical formula of two-dimensional downward flame spread rate over PMMA through a heat transfer model, which was proved to predict flame spread rate accurately. Mamourian et al. [11] conducted experiments on flame spread down over PMMA samples with various ratios of sample width to thickness and revealed that flame spread rate was closely related to sample dimensions. Experimental investigations were only concerned with downward flame spread over solid combustibles in a 2-D situation, where sample was hypothesized to be infinite along the sample width and pyrolysis front was linear and uniform. Very few papers [2,12,14] have addressed the flame spread over uninhibited solid materials, where flame spread was unconstrained and should be considered three-dimensional (3-D).

Flame height is another important parameter in estimating fire behavior. A number of experiments had been conducted on wall fires, to investigate the dependence of flame height on various parameters [20,21,22,23,24]. Delichatsios [20,21] proposed that flame height was independent of stoichiometry and gave a non-dimensional correlation of flame height with total heat release rate per unit width for a buoyant diffusion fire. Later, several studies [22,23] were carried out to optimize the flame height correlation. A consistent conclusion is that the correlation between flame height and heat release rate is related to the magnitude of the fire source. A power-law dependence of flame height on mass burning rate is also proposed and verified. Recently, Gollner et al. [24] gave a specific correlation of flame height with heat release rate for both laminar and turbulent wall fires, and proposed that the theoretical exponent of heat release rate was within the range of 2/3 and 4/3.

A literature review reveals that few investigations have been performed on a 3-D downward flame spread. The geometrical size of solid combustibles in actual use is not sufficiently wide, and the sample is more likely to be in an uninhibited condition. Thus, dominant mechanisms of 3-D downward flame spread need more experimental study. Heat transfer at the leading edge of the flame is enhanced since the stand-off distance is smaller and oxygen supply is rich there [2,7,12,14]. Measured results also clarified this point of view [7]. Therefore, the models of heat transfer for the leading edge and burning region are different, needing separate treatment as a consequence. Ambient pressure is also one of the most important and the most studied factors influencing the flame spread behavior [6,13,25]. In fixed boundary conditions, pressure has a significant influence on heat and mass transfer processes, which in turn will change the flame spread rate. However, pressure effects are not involved in downward flame spread over solid slab in free boundary conditions.

Thus, a series of experiments were conducted to investigate the effects of sample dimension and ambient pressure on downward flame spread over uninhibited PMMA slabs in this paper. PMMA was chosen as the fuel due to its common use in flame spread tests. Experiments of downward flame spread over PMMA with various dimensions were conducted at five locations with different altitudes. Experimental results will be compared with theoretical predictions to test the accuracy of the model.

## 2. Experimental

**Figure 1:**
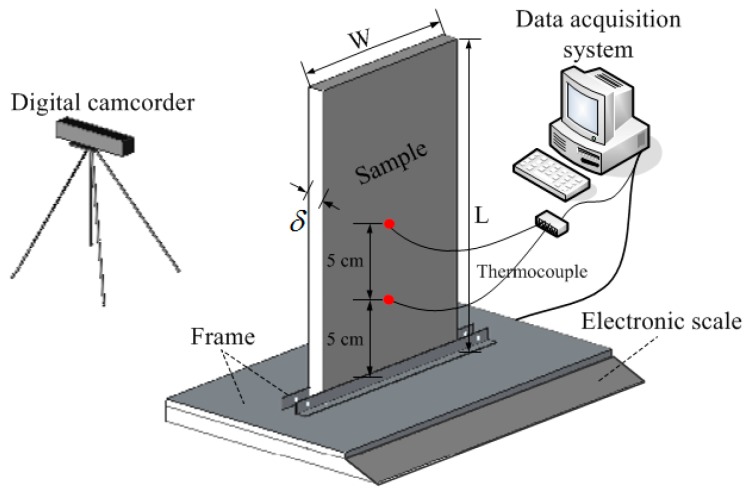
Schematic illustration of the experimental apparatus.

A schematic of the experimental apparatus is shown in Figure 1. Samples used are pure and transparent PMMA slabs, with density $\rho_{s}$ of 1.18 g/cm^3^. A series of experiments were conducted on samples with different dimensions. Three sample thicknesses (δ) were involved, 2, 5, and 10 mm; sample width (*W*) ranged from 3 to 18 cm with an interval of 3 cm; and the length (*L*) of the sample was 35 cm, long enough to reach a steady stage. It should be noted that there is no strip along the specimen which means that sample is absolutely free.

In order to record the mass loss history of PMMA, samples were mounted vertically on an electronic scale with a resolution of ±0.01 g. Two K-type thermocouples with a diameter of 0.5 mm were fixed close to the sample to measure the flame temperature. Samples were ignited at the top end by a linear methane diffusion flame. After ignition, behaviors of spreading flame and the profile of the burning sample were recorded with a digital camera at 30 fps located in front of the sample. Each test was repeated at least three times to ensure repeatability.

Experiments were conducted in five cities in China with different altitudes, Hefei, Xining, Geermu, Lhasa and Yangbajain. The ambient temperature and pressure at each altitude is provided in Table 1. Ambient temperature hardly changes with location, and is assumed to be negligible in this study. It should be noted that oxygen volume concentration at different altitudes is almost the same, which means that ambient pressure plays a significant role at plateau in this study.

A visual measurement method was employed in this study to obtain the flame spread rate, flame height and flame area using the recorded videos [14,24]. A total of 500 frames captured by KMPlayer from the video recorded by front-view camera during steady-state stage were imported into MATLAB, and were processed into binary images based on the gray-scaled pixel value. Flame spread rate was obtained after dividing the displacement of the flame front in steady-state stage by the selected time period. The mean value of flame area divided by the sample width was considered the averaged flame height.

## 3. Results and Discussion

**Figure 2:**
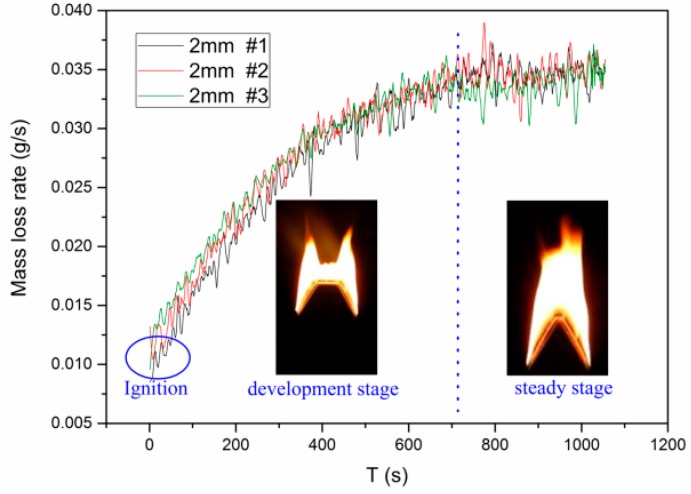
Mass loss rate histories and recorded flame profiles for 2 mm sample in 102 kPa. The flame spread process is divided into three stages: (1) ignition stage; (2) development stage; (3) steady-state stage.

Due to the enhanced convective heat transfer and sufficient oxygen supply, flame at the edge of each side spreads faster than center area after ignition [12,26]. As a result, the profile of flame front changes constantly in the initial period, before reaching a steady-state stage. Flame spread rate, profile of pyrolysis front and flame shape are invariable in this steady-state stage. It should be noted that the duration in different cases is indefinite, half an hour to an hour. In addition, the duration of the development stage changes regularly with sample dimension and pressure. As the sample dimension increases, including both width and thickness, the duration of the development stage increases. The time of development stage decreases as pressure increases. Figure 2 shows the mass loss rate history and recorded images in different burning stages for three repeated tests of 2 mm slabs in Hefei. It is seen that pyrolysis front in the steady-state stage appears as a flipped “V” shape, different from that in the case of 2-D downward flame spread [10,11] where the pyrolysis front is linear and straight in the whole spreading period. Unless specifically noted, all parameters in the text below are defined for the steady-state stage.

### 3.1. Prediction of Flame Spread Rate

**Figure 3:**
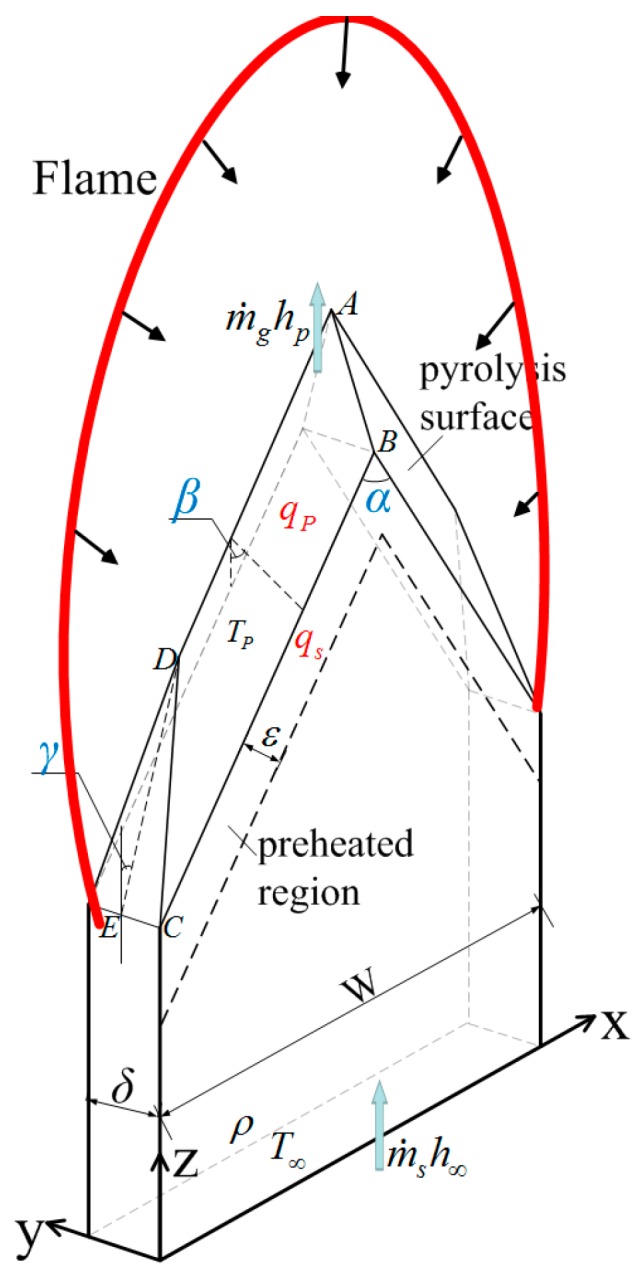
Sketch diagram of the theoretical model in the steady-state stage. Heating region in the model includes two parts, preheated region and pyrolysis surface. Total heat fluxes at preheated region and pyrolysis surface are *q_s_* and *q_p_*, respectively. α, β and γ are characteristic angles that emerged on sample residue in the steady-state stage. ${\dot{m}}_{g}h_{p}$ and ${\dot{m}}_{s}h_{\infty }$ refer to the variation of enthalpy of gas phase and solid phase. *T_p_*, $T_{\infty }$ and ε are pyrolysis temperature, ambient temperature and preheated length, respectively.

**Figure 4:**
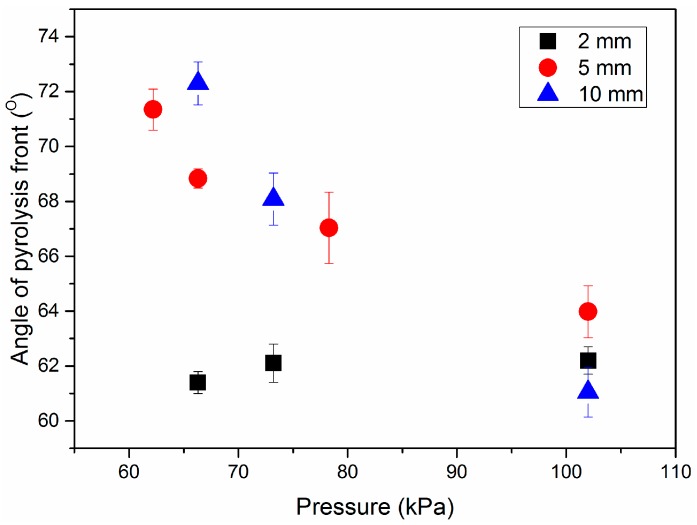
Angle of pyrolysis front (α) with respect to ambient pressure for different sample thicknesses (δ).

Our previous study [14] has built a predictive model of 3-D downward fire spread over PMMA in free conditions, as shown in Figure 3. This model is proved to be accurate in predicting flame spread rate under different sample thicknesses and widths. α, β, γ are characteristic angles that emerged on sample residue in the steady-burning stage and they are unchanged with sample width according to the experimental results and related studies [2,12,14]. α is the angle of pyrolysis front along the direction of width and is directly linked to the flipped “V” shape. Figure 4 shows the averaged values of α for three sizes of PMMA slabs at different pressure environments. Solid points in the figure denote the mean values of α for sample with different widths. It is noticed that the variation tendency of α with pressure is different for the three samples. In the case of the 2 mm thick sample, α is almost a constant. In contrast, for samples with thicknesses of 5 and 10 mm, it decreases significantly with increasing pressure. In addition, it can be seen that the variation of α with pressure is much more evident for the thicker sample. According to the measured results, β and γ are insensitive with pressure, which rarely changes, within two degrees. Mean values of β and γ are 20.6°, 13.2°, respectively, within the error in 10%.

Extensive works indicate heat transfer from flame to the virgin fuel through the gas phase is the most significant in downward flame spread over the surface of thin PMMA [6,25]. Thus, convective heat transfer through gas phase is considered the dominant mode of heat transfer in deciding flame spread rate in this study, as reported in previous studies [4,10,13,18,25,27]. Samples considered in this study are thermal intermediate according to Pello’s theory [6,25], which means heat transfer through solid phase has certain effects on flame spread process. However, heat delivered from burning solid to flame front (pyrolysis front) is hard to measure or calculate accurately. Thus, the whole sample is taken as a control volume and the energy balance on the whole sample is considered in this study. This method is verified to be able to predict downward flame spread rate [10,14]. Since the heat delivered to the pyrolysis region and the region ahead of it (preheated region) are very different, their heat transfer processes will be considered individually.

At the leading edge of the flame front corresponding to the preheated region in Figure 3, boundary layer approximation is not applicable as the characteristic length (the length of preheated region, ε, is approximately 2 mm [25]) is rather small. The calculated Grashof number *Gr* is less than 10, which means that buoyancy induced by natural convection is of the same order of magnitude as viscous force [28]. In other words, gas velocity in the gaseous thermal region at preheated region is negligible. Thus, thermal conduction becomes the unique important heat transfer model in gas-phase [28], which may be roughly estimated as

(1) $$q_{s}^{\prime \prime }=k_{g}\frac{T_{f}-T_{p}}{l}$$

where *T_f_* and *T_p_* are flame temperature and pyrolysis temperature, respectively; *k_g_* is the thermal conductivity; *l* is the normal distance from flame to preheated surface, which may be denoted by the thermal diffusion length $\bar{\delta }$ at the leading edge [8,15]. Based on the balance between convection and conduction terms in energy equation under naturally convective flow [29,30], the thermal diffusion length can be given by

(2) $$\bar{\delta }=\frac{\phi }{U_{ref}}$$

where ϕ and $U_{ref}$ are the thermal diffusion coefficient and the reference velocity at the leading edge. $U_{ref}$ is induced flow velocity due to density variation near the flame which can be equated from conservation equations of buoyancy and inertia force [15,29,30] as

(3) $$U_{ref}={\left(\frac{g(\rho_{\infty }-\rho_{f})\phi }{\rho }\right)}^{1/3}$$

where $\rho_{\infty }$ and $\rho_{f}$ are the density of air at ambient temperature and flame temperature, respectively; ϕ and ρ in Equations (2) and (3) denote the quantities evaluated at an arithmetical average of ambient and adiabatic flame temperatures. Rearranging Equations (1)–(3), heat flux transferred to the preheated region is estimated as follows:

(4) $$q_{s}^{\prime \prime }=k_{g}(T_{f}-T_{p}){\left[\frac{g(\rho_{\infty }-\rho_{f})}{\rho }\right]}^{1/3}\phi^{-2/3}$$

Experimental results reveal that flame temperature hardly changes with altitude, remaining approximately constant at 900 °C. Analogously, Liang et al. [31] found the temperature of burning PMMA was unchanged with altitude and gave the explanation that the combined effect of low oxygen concentration and less soot formation led to the invariable flame temperature with pressure. In addition, pyrolysis temperature *T_p_* (approximately 400 °C) and thermal conductivity of gas flow *k_g_* are insensitive to pressure within the normal range of pressure [13,28].

Based on ideal gas law, $\rho_{\infty }$, $\rho_{f}$ and ρ are linearly proportional to ambient pressure. The term in the brackets in Equation (4) is invariable with pressure as the counterbalance effect. Thus, it can be deduced that $q_{s}^{\prime \prime }$ is only related to thermal diffusivity ϕ according to Equation (4). Based on the thermal transfer theory, thermal diffusivity is estimated to be related with thermal conductivity, density of flow and specific capacity [28,32] as

(5) $$\phi =\frac{k_{g}}{\rho c_{p}}$$

where *c_p_* is specific capacity, an inherent parameter. According to the ideal gas law, air density is proportional to the ambient pressure. Therefore, the dependence of heat flux in the preheated region on pressure can be simplified by inserting Equation (5) into Equation (4) as

(6) $$q_{s}^{\prime \prime }\propto \rho^{2/3}\propto P^{2/3}$$

Pyrolysis surface in this study is inclined. The orientation is defined as its inclination angle from the vertical, which is denoted by θ. The value of θ can be interpreted from the simplified model, and its resulting expression is presented in Equation (7),

(7) $$\cos\theta =\sqrt{1-(\cos(\alpha /2)\sin(\beta /2){)}^{2}}$$

For an inclined surface, the gravitational acceleration in calculating the Grashof number must be replaced by its streamwise component gcosθ [3,33,34,35,36,37]. As a result, the modified Grashof number $Gr^{*}$ is given by

(8) $$Gr^{*}=\frac{g\cos\theta \times \psi (T_{f}-T_{p})L^{3}}{v^{2}}$$

where ψ is the volume thermal expansion coefficient; *L* is characteristic length along fire plume, which can be estimated as the fire length of unit width, 1/sin(α/2) in this study; and v is kinematic viscosity. The characteristic streamwise length is on the order of centimeter. As a result, the order of magnitude of the modified $Gr^{*}$ is $10^{4}$ according to Equation (8), which suggests that the approximation of laminar convective boundary layer is valid here [28]. The convective heat transfer coefficient *h* using the assumption of boundary layer can be evaluated by Nusselt number *Nu*, thermal conductivity and characteristic length [28] as

(9) $$h=\frac{Nu\times k_{g}}{L}$$

For laminar flow, *Nu* is given by [28]:

(10) $$Nu={\left(\frac{Gr^{*}}{4}\right)}^{1/4}\times g(\mathrm{Pr})$$

where g(Pr) is the function of Prandtl number Pr, 0.73 in air environment [28]. Thus, convective heat coefficient is obtained from Equations (9) and (10)

(11) $$h=k_{g}{\left[\frac{g\psi (T_{f}-T_{p})}{4v^{2}}\right]}^{1/4}g(\mathrm{Pr})L^{-1/4}\cos\theta^{1/4}$$

Kinematic viscosity v in Equation (11) is related to flow density, v=η/ρ, where η is dynamic viscosity which is invariable with pressure. Convective heat flux $q_{P}^{\prime \prime }$ is proportional to convective heat transfer coefficient and the temperature gradient, and thus can be written as

(12) $$q_{P}^{\prime \prime }=h(T_{f}-T_{p})$$

The correlation of convective heat flux at pyrolysis surface can be derived from Equations (11) and (12) as

(13) $$q_{P}^{\prime \prime }=k_{g}{\left[\frac{g\psi }{4{\left(\eta /\rho \right)}^{2}}\right]}^{1/4}{\left(T_{f}-T_{p}\right)}^{5/4}g(\mathrm{Pr})L^{-1/4}\cos\theta^{1/4}$$

As pointed out in previous sections, *L* and cosθ are changed with pressure. The variation of $L^{-1/4}\cos\theta^{1/4}$ in Equation (11) is rather small, within 3%, which is negligible here. Apart from density of the fire flume, other parameters in Equation (13) can be considered to be invariable with pressure. Thus, Equation (13) can be simplified as

(14) $$q_{P}^{\prime \prime }\propto \rho^{1/2}\propto P^{1/2}$$

Heat flux distributions at the pyrolysis surface and preheated region in Hefei were deduced in our previous study, which are 20.6 and 73.3 kW/m^2^, respectively [14]. According to Equations (6) and (14), distributions of heat flux in other four pressure environments can be derived through Equations (15) and (16) as

(15) $$q_{s}^{\prime \prime }=73.3{\left(\frac{P}{102}\right)}^{2/3},$$

(16) $$q_{P}^{\prime \prime }=20.6{\left(\frac{P}{102}\right)}^{1/2},$$

where the unit of *P* is kPa.

**Figure 5:**
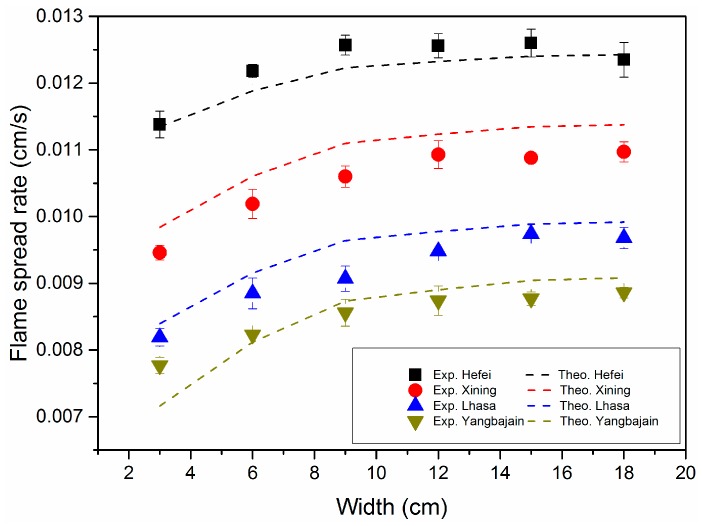
Comparisons between experimental and theoretical flame spread rates over a 5 mm slab for different widths under different pressure environments.

**Figure 6:**
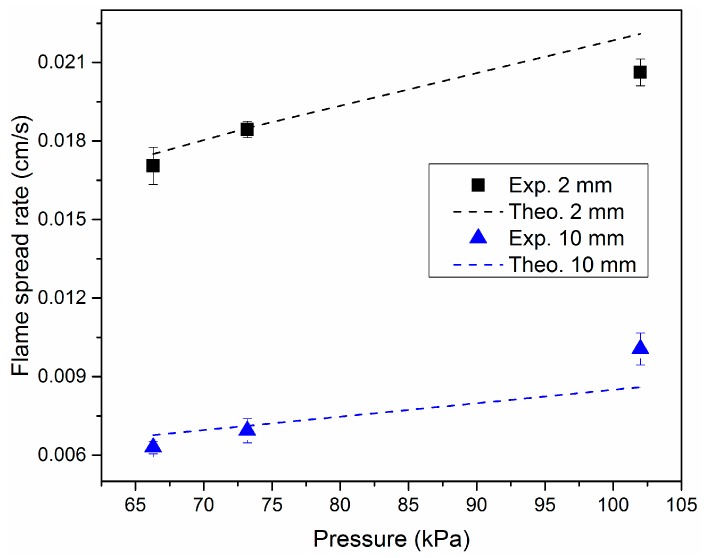
Comparisons between experimental and theoretical flame spread rates over 2, 10 mm slabs under three different pressure environments.

In our previous study [14], a calculating formula was proposed to predict the steady flame spread rate based on heat transfer theory and energy conservation equation,

(17) $$V_{f}=4\frac{\left[\frac{\delta^{2}\cot\frac{\beta }{2}}{8\sin(\frac{\alpha }{2}-\gamma )}+\frac{W\delta }{4\sin\frac{\alpha }{2}\sin\frac{\beta }{2}}-\frac{\delta^{2}\cot\frac{\beta }{2}\sin\gamma }{8\sin\frac{\alpha }{2}\sin(\frac{\alpha }{2}-\gamma )\sin\frac{\beta }{2}}\right]\times q_{P}^{\prime \prime }+(\frac{\delta }{2}+\frac{W}{2\sin\frac{\alpha }{2}})\varepsilon \times q_{s}^{\prime \prime }}{(h_{\deg}+c_{P}(T_{p}-T_{\infty }))\times \rho_{s}W\delta }$$

where $h_{\deg}$ is the heat of degradation of solid PMMA. Plugging the measured characteristic angles and the calculated heat fluxes into Equation (17), flame spread rates for different-sized samples in five pressure environments can be estimated. Figure 5 and Figure 6 show the comparisons between measured and calculated flame spread rates for different dimensions and different pressure environments. The solid points in two figures denote experimental results for three repeated tests and the dashed lines represent the calculated results from Equation (17). It is seen that predicted values from theory agree well with experimental results for the different-sized samples this paper concerns in different pressure environments. Flame spread rate increases first and then tends to be unchanged with sample width. Compared with sample width, pressure and sample thickness seem to have greater influence on the flame spread rate.

**Figure 7:**
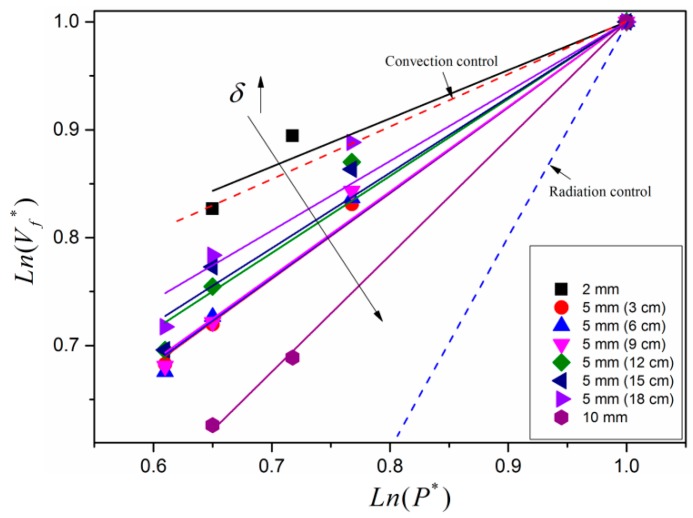
Fitted relationships between flame spread rate and pressure for different sized samples. The horizontal and vertical coordinates are the values of non-dimensional pressure ($P^{*}$) and non-dimensional flame spread rate ($V_{f}{}^{*}$) after logarithm.

According to the scale modeling of surface combustion and flame spread over solid combustibles, a power-law dependence of burning rate or flame spread rate on pressure is obtained [13,25,38]. In order to take a comprehensive consideration of three different thickness samples, a dimensionless treatment is adopted here. Figure 7 shows the exponential fitted relationships between normalized flame spread rate and normalized pressure. The fitted parameters for different sized samples are presented in detail in Table 2. The superscript * denotes the relative quantity, which is equal to the actual value at low pressure environment divided by the value at normal pressure (102 kPa), $V_{f}{}^{*}=V_{f}/V_{Hefei}$, $P^{*}=P/P_{Hefei}$. The solid points denote experimental results, and the solid lines are fitted results for experimental results with the same dimension. It is seen that the exponent changes regularly with the sample dimension. As sample thickness increases, the fitted exponent increases as well, ranging from 0.45 to 1.08. In contrast, for sample with the same thickness, the fitted exponent is nearly invariable. It can be deduced that width effect on heat transfer mechanism at each altitude is marginal. Two dashed lines represent correlations of convection-controlling and radiation-controlling flame spreads over solid surface with pressure in a 2-D case, and their slopes are 0.5 and 2 [3,18,19,39], respectively. In this study, convective heat delivery through gas-phase is dominant for different sized samples. Furthermore, the effect of pressure on thermal feedback mechanism at pyrolysis surface and preheated region for different sized samples is taken as the same. However, the dependence of flame spread rate on pressure appears to change significantly with sample thickness. This discrepancy may be explained by the fact that the effect of angle of pyrolysis front on flame spread rate plays a significant role in the 3-D case.

### 3.2. Prediction of Angle of Pyrolysis Front

In this study, the relationship among flame spread rate, angle of pyrolysis front, sample dimensions and thermophysical parameters of solid PMMA is presented in Equation (17). Compared with pressure, sample width has little effect on angle of pyrolysis front and the heat delivery mechanism according to the experimental results. As a result, the angle of the pyrolysis front is invariable with sample width. When sample width is much larger than thickness, Equation (17) reduces to

(18) $$V_{f}=4\frac{\frac{W\delta }{4\sin\frac{\alpha }{2}\sin\frac{\beta }{2}}\times q_{P}^{\prime \prime }+\frac{W}{2\sin\frac{\alpha }{2}}\varepsilon \times q_{s}^{\prime \prime }}{(h_{\deg}+c_{p}(T_{p}-T_{\infty }))\times \rho_{s}W\delta }$$

Thus, the sine of α/2 can be derived from Equation (18) as follows:

(19) $$\sin\frac{\alpha }{2}=[\frac{q_{p}^{\prime \prime }}{\sin\frac{\beta }{2}(h_{\deg}+c_{p}(T_{p}-T_{\infty }))\rho_{s}}+\frac{2\varepsilon \times q_{s}^{\prime \prime }}{(h_{\deg}+c_{p}(T_{p}-T_{\infty }))\times \rho_{s}\delta }]/V_{f}$$

The sum of the two terms in brackets in Equation (19) denotes flame spread rate normal to the pyrolysis front (2-D). It can be indicated that the value of sin(α/2) equals to the ratio of flame spread rate normal to the pyrolysis front to the downward flame spread rate. The first term in the brackets tends to be much larger than the second term, especially for larger thickness. Thus, the relationship between the sum of the two terms in bracket and pressure is simplified as that of the first term with pressure, which means that flame spread rate normal to the pyrolysis front is proportional to pressure with an exponent of 0.5 approximately. Similarly, Fernandez-Pello et al. [19] proposed that 2-D opposing flame spread rate over a cylinder of PMMA for diameters ranging from 0.8 to 12.7 mm was proportional to environmental and thermal physical parameters as

(20) $$V_{2D}\propto exp[-E_{g}/(RT_{f})]Y_{O}{}^{2}P^{0.5}U_{\infty }{}^{-1.5}$$

where $E_{g}$, R, $Y_{O}$, and $U_{\infty }$ are activation energy for fuel pyrolysis, universal gas constant, mass fraction of oxygen and ambient velocity, respectively. It shows that flame spread rate in a 2-D case is exponentially proportional to pressure with an exponent of 0.5. Thus, Equation (19) reduces to

(21) $$\sin\frac{\alpha }{2}=\frac{C_{1}P^{0.5}}{V_{f}}$$

where $C_{1}$ is a parameter invariable with pressure. As pointed out in the previous section, flame spread rate $V_{f}$ is exponentially proportional to pressure for sample with the same thickness. Thus, the correlation of sin(α/2) with pressure is given by

(22) $$\sin\frac{\alpha }{2}=\frac{C_{1}P^{0.5}}{C_{2}P^{n}}$$

where $C_{2}$ is a parameter which is variable with sample thickness; *n* is presented in Table 2.

**Figure 8:**
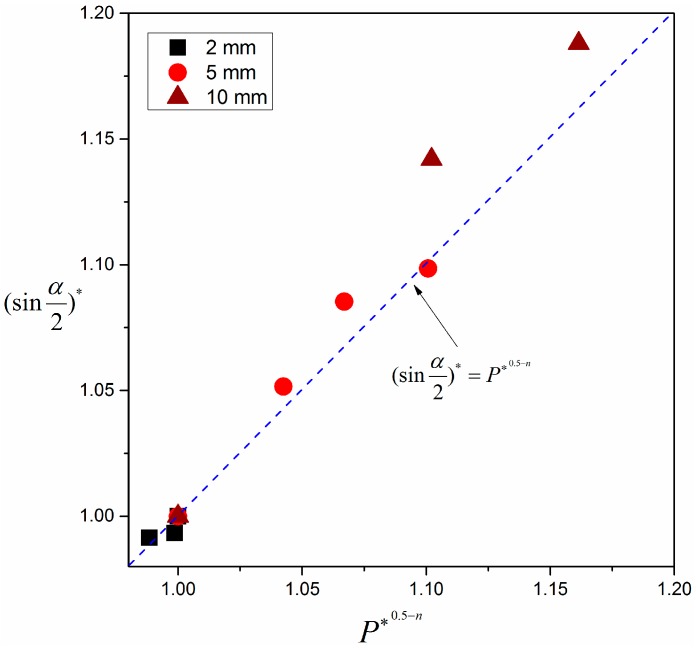
Non-dimensional dependence of sin(α/2) on pressure.

In order to get rid of the effect of sample dimension, a dimensionless variation tendency of the angle of the pyrolysis front with pressure is derived as

(23) $${\left(\sin\frac{\alpha }{2}\right)}^{*}=P^{{*}^{0.5-n}}$$

where the superscript * denotes the relative quantity, which is equal to the actual value in various pressure environments divided by the value at normal pressure (102 kPa) for the same experiment. Figure 8 shows the comparison of the correlation between angle of pyrolysis front and pressure for experimental and theoretical results. The colored points denote averaged experimental results over sample width; the blue dashed line is the predicted tendency from Equation (23). It shows that prediction from Equation (23) coincides fairly well with the experimental result.

### 3.3. Prediction of Averaged Flame Height

Profile of burning residues in the steady-state stage appears as a flipped “V” shape, which in turn forms a non-linear flame front. In order to remove the influence from the non-linear flame front, averaged flame height denoted by the ratio of flame area to sample width is adopted in this study. Previous studies revealed that flame height depended closely on the heat release rate for wall fires [20,21,22,23,24]. Its specific relationship is concerned with the status of fire plume. The fire plume in this study is assumed to be laminar according to Equation (8), which means that oxygen reaches the flame region mainly by molecular diffusion. Gollner et al. [24] gave the relationship between flame height and burning rate per unit width in laminar wall-fires as

(24) $$H_{f}=\frac{{{\dot{m}}^{\prime }}^{4/3}}{{\left(\rho_{\infty }\rho_{f}f^{2}D\sqrt{g}\right)}^{2/3}}$$

where *f*, *D* are the mass of fuel required to react with a unit mass of air and molecular diffusion coefficient of gaseous fuel, respectively. Mass burning rate per unit width can be denoted as $\dot{m}/[W*\sin(\alpha /2)]$ in this study. According to the mass transfer theory, the diffusion coefficient is relevant to system pressure and temperature as

(25) $$D\propto P^{-1}T_{f}{}^{3/2}$$

The power-law dependence of flame height on mass loss rate can be simplified by plugging the expressions of mass burning rate per unit width and the diffusion coefficient into Equation (24) as follow

(26) $$H_{f}\propto {\left[\frac{\dot{m}}{WP^{1/2}}\right]}^{4/3}$$

**Figure 9:**
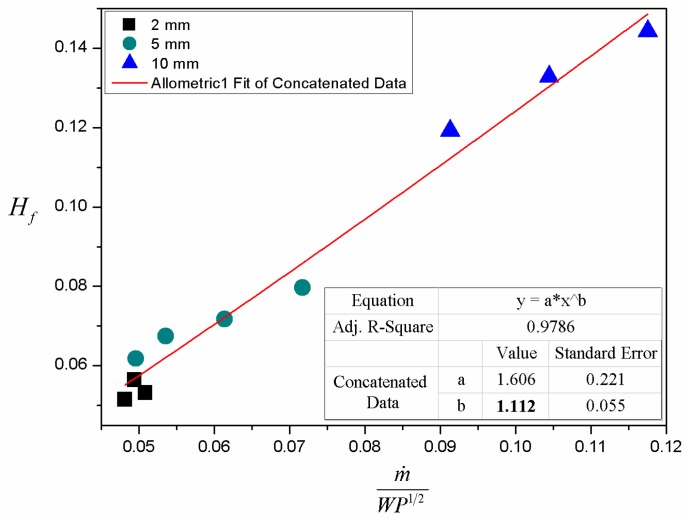
Relationship between averaged flame height and burning rate: $H_{f}$ to $\frac{\dot{m}}{WP^{1/2}}$. The red line is an exponential fit to the experimental data.

Figure 9 shows the fitted correlation of averaged flame height with mass burning rate and pressure. The solid points represent averaged data over width, as the averaged values of flame height and mass burning rate per unit width change little with sample width according to the experimental results. The index derived from the experimental data is 1.112 and is close to the theoretical result (4/3) [18], which indicates that the correlation of flame height on heat release rate is valid in 3-D downward flame spread as well. A smaller fitted exponent in comparison with theoretical result may result from experimental error. In addition, the fire plume in the experiment is not completely laminar. It means that air entrainment and eddy contribute the air supply to fire plume as well, which in turn weakens the dependence of flame height on mass burning rate.

Mass burning rate is proportional to flame spread rate in the steady-state stage:

(27) $$\dot{m}=\rho W\delta V_{f}.$$

**Figure 10:**
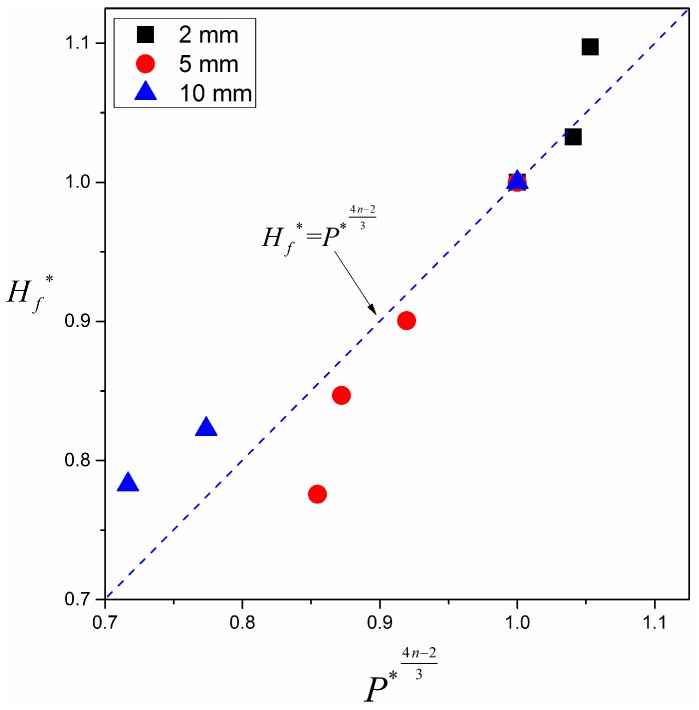
Non-dimensional dependence of flame height on pressure.

Flame spread rate is exponentially proportional to pressure, $V_{f}\propto P^{n}$, and the value of *n* is presented in detail in Table 2. Thus, dimensionless correlation of flame height with pressure is given by plugging Equation (27) into Equation (26),

(28) $$H_{f}{}^{*}=P^{{*}^{\frac{4n-2}{3}}}$$

where the superscript * denotes the relative quantity, which is equal to the actual value in various pressure environments divided by the value at normal pressure (102 kPa) for the same experiment. Figure 10 shows the comparison of flame height between experimental results and theoretical predictions from Equation (28). Solid points denote averaged experimental values over different widths since flame height is unchanged with pressure. It can be seen that experimental results of flame height are in reasonable agreement with theoretical predictions as well. As sample thickness increases, the exponent n increases according to previous derivation. Thus, the exponent of non-dimensional pressure in Equation (28) increases as well.

## 4. Conclusions

Downward flame spread tests were conducted on PMMA with different dimensions at five different altitudes. The main properties of the 3-D downward flame spread that were studied were flame spread rate, angle of pyrolysis front and flame height. Their relationship and variation tendency with pressure were proposed based on experimental results and theoretical interpretation. The main results are summarized as follow:

1. Heat fluxes at the pyrolysis surface and preheated region increase exponentially with pressure, with exponents of 1/2 and 2/3, respectively. Through the established 3-D theoretical model and heat flux distributions, flame spread rate in different pressure environments was predicted successfully. In addition, it was found that flame spread rate was exponentially proportional to pressure, approximately. The fitted exponent increases with sample thickness. However, it merely changes with sample width.
2. The changing trends of angle of the pyrolysis front with pressure for samples with different thicknesses are distinctly different. For the 2 mm sample, the angle of the pyrolysis front hardly changed with pressure. In contrast, for a larger sample width, this angle decreases significantly with increasing pressure, which in turn will increase the length of the burning region. Through the derived correlation of 3-D flame spread rate, a normalized formula for the angle of the pyrolysis front with pressure was proposed, ${\left(\sin\frac{\alpha }{2}\right)}^{*}=P^{{*}^{0.5-n}}$, which coincided well with experimental results in this study.
3. A power-law dependence of averaged flame height on mass burning rate, sample dimension and ambient pressure in downward flame spread was presented based on laminar diffusion theory and confirmed through experimental results. The fitted exponent is 1.11, basically consistent with the theoretical result. In addition, a normalized correlation of flame height with pressure was proposed as well, $H_{f}{}^{*}=P^{{*}^{\frac{4n-2}{3}}}$.

This paper gives a method to help estimate characteristics of 3-D downward flame spread in different pressure environments. Additional work on flame spread under complex boundary conditions will be needed to understand the mechanisms of 3-D flame spread.

## Figures and Tables

**Table 1:** Atmosphere parameters at five different altitudes.

Location	Altitude (m)	Ambient Pressure (kPa)	Ambient Temperature (°C)
Hefei	30	102	11–15
Xining	2295	78.3	9–13
Geermu	2800	73.2	7–12
Lhasa	3650	66.3	9–13
Yangbajain	4300	62.2	7–10

**Table 2:** Fitted parameters corresponding to Figure 7.

Sample Dimension (δ−W)	Slope (*n*)	Standard Error	Adjusted R-Square
2 mm–5 cm	0.45	0.041	0.954
5 mm–3 cm	0.79	0.018	0.995
5 mm–6 cm	0.79	0.026	0.989
5 mm–9 cm	0.79	0.029	0.986
5 mm–12 cm	0.71	0.045	0.963
5 mm–15 cm	0.70	0.045	0.962
5 mm–18 cm	0.65	0.050	0.946
10 mm–3 cm	1.08	0.012	0.999
